# Supplementary material for: Prevalence, clinical correlates and outcomes of cardiorenal anemia syndrome among patients with heart failure attending tertiary referral hospital in Dodoma, Tanzania: A protocol of a prospective observational study
Source: PLoS One. 2024 Jan 2;19(1):e0292764. doi: 10.1371/journal.pone.0292764 (PMC10760854; doi:10.1371/journal.pone.0292764)
Supplement: Attachment — Submitted filename: Response to reviewers.docx [file pone.0292764.s002.docx]

# **Response to reviewers**

**Dear Editor-in-chief,**

**PLOS ONE.**

We express our cordial gratitude for the valuable and constructive assessment and review of our paper. The reviewer’s comments have helped us in deed to further strengthen the quality of our work. With utmost care, we are incorporating all the suggestions/corrections as proposed by the reviewers.

In the following, we address the concerns point by point:-

**Comment 1**:-

The PLOS ONE publication guidelines are not followed and adhered.

**Response 1**: The manuscript has been updated so as, to strictly adhere to the recommended guidelines, including:-

- Superscripting of the authors’ affiliations (**refer**, page number 1, line 9 to 12)
- Omission of the full physical address and use of corresponding author’s initials (**refer**, page number 1, line 15)
- Styling, formatting and font size of main document as per PLOS ONE publication guideline (**refer**, page numbers 5, 12, 16, 18 and 19, lines 104, 106, 195,310, 355, 359)
- Omission of incomplete reference list and updating the complete reference list as per guideline (**refer**, page numbers 20 to 25, lines 377 to 544)
- Updating tables’ captions and citation of the tables was done (**refer**, page numbers 8 to 11, lines 177, 178, 184, 185, 191, 192)
- Spacing of words (**refer**, page number 14, line 259)
- Page breaking (**refer**, page numbers 16, 18 and 19, lines 310, 353, 359)

**Comment 2**:-

Difficulties in assessment of the third objective of the study since the power of the study is not strong enough. Sample size estimation favors the first objective and it will be less likely to assess the third objective.

**Response 2:** The manuscript was updated as per PLOS ONE publication guideline as follows:-

- Re-calculation and re-estimation of sample size and increase on sample size was done so as to strengthen the power of our study. Also, reference article used for estimation of sample size was changed (**refer**, page numbers 6 and 12, lines 131, 143, 147, 148, 153, 201)
- Modification and re-writing of the third objective of the study was done by including all-cause mortality and heart failure hospitalization after passing through composite endpoints for heart failure (HF) trials as per articles recommended by reviewers. Additional referencing was done (**refer**, page number 7, lines 164 to 172)

Sincerely,

**John Meda**

**Corresponding author.**
